# Supplementary material for: Prognostic accuracy of the CMPMIT-ICD-10, APACHE Ⅱ, SOFA, ISS, and AIS for in-hospital death among patients with traumatic hemorrhagic shock
Source: PLoS One. 2026 Feb 5;21(2):e0340159. doi: 10.1371/journal.pone.0340159 (PMC12875474; doi:10.1371/journal.pone.0340159)
Supplement: S1 File — (DOCX) [file pone.0340159.s001.docx]

**Table S1** the ICD-10 code of trauma areas and complications for CMPMIT-ICD10

| **Trauma regions and complications** | **ICD-10-CM code** |
| --- | --- |
| Head |  |
| A1 | S00.0; S01.0; S09.1 |
| A2 | S02.0; S02.9; S04; S06.0; S07.0; S08.0; S09.0 |
| A3 | S02.1; S06.1; S06.301; S06.302; S06.381; S06.382; S06.6; S06.701; S06.702; S06.705; S06.706; S06.805; S06.809; s06.810; S06.811; S06.812; S06.813; S06.902; S06.901 |
| A4 | S06.201; S06.202; S06.211; S06.221; S06.231; S06.232; S06.233; S06.281; S06.282; S06.311; S06.321; S06.331; S06.341; S06.4; S06.5; S06.703; S06.707; S06.802; S06.804; S06.807; S06.801; S06.803; S06.806 |
| A5 | S06.212; S06.291; S06.332; S06.342; S06.704; S06.708; S06.808; S06.903; S07.1; S08.901 |
| Maxilloface |  |
| B1 | S00.1; S00.2; S00.3; S00.4; S00.5; S00.7; S00.8; S00.9; S01.1 S01.2; S01.3; S01.4; S01.5; S01.7; S01.8; S00.9; S02.2; S02.5; S02.6; S03.1; S03.2; S03.3; S03.4; S03.5; S05.0; S05.1; S05.3; S05.4; S05.8; S05.9; S09.2; S09.8 |
| B2 | S02.3; S02.4; S02.7; S02.8; S03.0; S05.2; S05.7; S05.5; S05.6 S08.1; S08.801 |
| - [Cervical region](javascript:%20void(0)) |  |
| C1 | S10.8; S10.9; S11.882; S11.901; S13.6; S14.4; S14.5; S15.2; S15.3 S16 |
| C2 | S10.002; S10.004; S10.003; S11.021; S10.001; S11.221 S10.005; S15.002; S10.1; S15.001; S11.011; S15.003; S11.101; S15.011 S11.211; S15.031; S11.881; S17.0; S11.811; S17.9; S11.821; S19.8; S12.8; S13.2; S13.5; S15.021; S15.1; S15.8 |
| [Thorax](javascript:%20void(0)) |  |
| D1 | S20; S21; S22.3; S23.2; S23.4; S23.5; S24.3; S29.0 |
| D2 | S22.2; S22.411; S22.421; S22.431; S22.401; S22.8; S22.9; S24.4 S24.5; S25.5; S25.8; S27.501; S27.601; S27.821; S27.831; S27.832; S27.841; S27.881 |
| D3 | S22.441; S22.5; S26.831; S25.1;S25.001; S26.882; S25.2; S27.321; S26.883; S25.301;S27.381; S26.881; S25.302; S27.401; S28.1; S25.401; S27.813; S25.7; S26.0; S26.811; S26.821; S26.901; S27.0; S27.1;S27.2; S27.312; S27.311; S27.402; S27.811; S27.812; S22.5; S26.831; S25.001;S26.882; S27.321; S26.883; S27.381; S26.881; S27.401; S28.1; S27.813 |
| Abdomen |  |
| E1 | S30; S31; S37.4; S37.881; S37.811; S39.0; S39.8 |
| E2 | S34.5; S34.6; S34.8; S35.504; S35.506; S35.508; S35.703; S35.8; S35.9; S36.011; S36.021; S36.001; S36.101; S36.111; S36.113; S36.121; S36.131; S36.181; S36.171; S26.201; S36.221; S36.231; S36.301; S36.491; S36.493; S36.401; S36.511; S36.521; S36.531; S36.541; S36.592; S36.593; S36.501; S36.601; S36.822; S36.811; S36.821; S37.01; S37.001; S37.021; S37.022; S37.102; S37.211; S37.201;S37.311; S37.321; S37.331; S37.382; S37.393 S37.301; S37.302 S37.5 S37.603 S37.821 S37.841 S37.831 S38.0 |
| E3 | S35.1; S35.0; S35.2; S36.041; S35.3; S34.151; S35.4;S36.211;S35.501;S36.412;S35.502; S37.031; S35.503; S37.22; S35.505; S35.507; S35.701; S35.702; S35.704; S35.705; S36.031; S36.081; S36.141;S36.202; S36.291; S36.292; S36.303; S36.403;S36.492; S36.494;S36.411; S36.502; S36.495; S36.591; S36.602; S36.603; S36.831; S37.023; S37.101; S37.281; S37.303; S37.304; S37.305; S37.381;S36.602;S36.601; S38.2; S38.1 |
| Spine |  |
| F1 | S23.3; S33.5; S33.7; S13.4; S12.0; S12.2; S12.901; S13.0; S12.904; S12.905; S13.12; S13.13; S13.14; S13.15; S13.16; S13.17; S13.18 S13.10; S14.2; S22.003; S22.006; S22.001; S22.011; S22.021; S22.031; S22.041; S22.051; S22.061; S23.0; S23.1; S24.201; S32.0; S32.821; S32.822; S32.824; S33.0; S33.1; S34.2; S34.4 |
| F2 | S12.1; S12.906; S12.903; S13.11; S14.0; S22.005; S22.007; S22.009; S22.1; S24.0; S32.702; S32.823; S34.0; S34.3; S14.12; S14.13; S14.1x7; S14.101; S24.12; S24.1x7; S24.101; S34.101; S34.103; S34.1x7; S14.111; S24.111; S34.102 |
| Limbs and pelvis |  |
| G1 | S40; S41; S43.4; S43.5; S43.6; S43.7; S45.202; S45.3; S46; S50; S51; S53.0; S53.1; S53.4; S44.0; S44.1; S44.2; S44.3; S44.4; S44.5; S54.001; S54.101; S54.201; S54.3; S55.0; S55.1; S55.2; S56; S60; S61; S62.5; S62.6; S62.7; S63.1; S63.4; S63.5; S63.6; S64; S65; S66; S33.6; S70; S71; S73.1; S74.2; S75.2; S76.0; S76.102; S76.103; S76.104; S76.2; S76.3; S80; S81; S83.401; S83.403; S83.411; S83.412; S83.421; S83.422 S83.502; S83.503; S83.51; S83.52; S83.602; S83.604; S83.605; S83.606; S83.607; S83.608; S83.601; S83.610; S84.2; S90; S91; S92.4; S92.5; S92.7; S93.1; S93.3; S93.4; S93.5; S93.6; S94; S95; S96 |
| G2 | S32.1; S32.2; S32.502; S32.501; S32.811; S33.201; S33.202; S73.0; S74.0; S74.1; S74.7; S74.8; S75.1; S75.7; S76.101; S76.7; S82; S82.1; S82.2; S82.3; S82.4; S82.5; S82.6; S82.8; S83.0; S83.1; S83.2; S83.3; S83.402; S83.43; S83.44; S83.501; S83.53; S83.54; S83.609; S83.603; S83.7; S84.0; S84.1; S84.7; S84.8; S87; S92.0; S92.1; S92.2; S92.3; S93.0; S93.2; S97; S42.0; S42.1; S42.2; S42.3; S42.4; S42.8; S43.0; S43.1; S43.2; S43.3;S45.0; S45.1; S45.201; S52.0; S52.1; S52.3; S52.2; S52.4; S52.5; S52.6; S53.2; S53.3; S44.7; S54.002; S54.102; S54.7; S55.7; S62.0; S62.1; S62.2; S62.3; S62.4; S63.0; S63.3; S67; S68.0; S68.1; S68.3; S68.2 |
| G3 | S42.7; S47; S48; S52.7; S57; S58; S68.4; S68.8; S32.3; S32.4; S32.503; S32.891; S32.892; S32.893; S32.831; S33.3; S33.4; S33.203; S72; S75.0; S75.8; S77; S82.7; S88; S98; S32.894; S32.895; S78 |
| Complications |  |
| Myocardial infarction | I20-25 |
| Congestive heart failure | I50 |
| Chronic renal failure | N17-N19 |
| Cerebrovascular diseases | I60-I69 |
| Hypertension | I10-I15 |
| Diabetes mellitus | E10-E14 |
| Chronic liver disease | K70-K77 |
| Malignant tumor | C00-C97 |
| Chronic lung disease | J40-J47 |
| Peptic ulcer | K25-28 |

**Table S2** General and clinical characteristics of the patients with traumatic haemorrhagic shock

| Characteristics | n= 420 |
| --- | --- |
| Male, n(%) | 233(55.5) |
| Age, years, median (IQR) | 60 (48–78) |
| Comorbidities, n(%) |  |
| Stroke | 33（7.9） |
| Coronary heart disease | 22（5.2） |
| Hypertension | 126（30） |
| Chronic obstructive pulmonary disease | 14（3.3） |
| Diabetes mellitus | 53（12.6） |
| Chronic kidney disease | 6（1.4） |
| Cancer | 29（6.9） |
| Causes of trauma, n(%) |  |
| Falling from height | 68（16.2） |
| Road trafﬁc accident | 168（40） |
| Falling from a standing position | 150（35.7） |
| Others (crush, stab, animal bite) | 34（8.1） |
| Main bleeding site, n(%) |  |
| Thoracic | 79（18.8） |
| Abdominal | 52（12.4） |
| Pelvic | 56（13.3） |
| Limbs | 165（39.3） |
| Others (blood vessels, skin, and soft tissue) | 68（16.2） |
| CMPMIT-ICD10 score, median (IQR) | 52（46-60） |
| APACHEⅡ score, median (IQR) | 18（14-22） |
| SOFA score, median (IQR) | 4（3-7） |
| ISS, median (IQR) | 20（14-29） |
| AIS score, median (IQR) | 10（7-15） |
| Acute respiratory distress syndrome, n(%) | 110（26.2） |
| Acute myocardial injury, n(%) | 207（49.3） |
| Acute liver injury, n(%) | 123（29.3） |
| Acute kidney injury, n(%) | 116（27.6） |
| Trauma-induced coagulopathy (n, %) | 61（14.5） |
| Hospital mortality (n, %) | 41（9.8） |
| Hospital LOS, days, median (IQR) | 17（11-25） |
| ICU LOS, days, median (IQR)) | 7（3-15） |

*IQR* Interquartile range, *CMPMIT-ICD10* China Mortality Prediction Model in Trauma based on the ICD-10-CM lexicon, *APACHEⅡ*Acute Physiology and Chronic Health Evaluation, *SOFA* Sequential Organ Failure Assessment, *ISS* Injury Severity Score, *AIS* Abbreviated Injury Scale, *LOS* Length of Stay, *ICU* Intensive Care Unit

**Table S3** AUCs for predicting in-hospital mortality among the five scores in patients with mechanical ventilation

|  | **Mechanical ventilation n=325** | | |
| --- | --- | --- | --- |
| **Variables** | **AUC** | **95%CI** | ***p* value** |
| CMPMIT-ICD10 | 0.8663 | 0.8101-0.9225 | 0.029 |
| APACHEⅡ | 0.8637 | 0.8048-0.9227 | 0.030 |
| SOFA | 0.7276 | 0.6340-0.8212 | 0.048 |
| ISS | 0.6007 | 0.4969-0.7046 | 0.053 |
| AIS | 0.5552 | 0.4523-0.6581 | 0.053 |

*CMPMIT-ICD10* China Mortality Prediction Model in Trauma based on the ICD-10-CM lexicon, *APACHEⅡ* Acute Physiology and Chronic Health Evaluation, *SOFA* Sequential Organ Failure Assessment, *ISS* Injury Severity Score, *AIS* Abbreviated Injury Scale

**Table S4** Best cutoff values, sensitivity, specificity, PPV, NPV, and accuracy of the five scoresin patients with mechanical ventilation

|  | **Mechanical ventilation n=325** | | | | | |
| --- | --- | --- | --- | --- | --- | --- |
| **Variables** | **Cutoff value** | **Sensitivity** | **Specificity** | **PPV** | **NPV** | **Accuracy** |
| CMPMIT-ICD10 | 64.5 | 0.800 | 0.814 | 0.376 | 0.967 | 0.812 |
| APACHEⅡ | 22.5 | 0.775 | 0.828 | 0.388 | 0.963 | 0.822 |
| SOFA | 7.5 | 0.625 | 0.751 | 0.260 | 0.934 | 0.735 |
| ISS | 34.6 | 0.350 | 0.902 | 0.333 | 0.908 | 0.834 |
| AIS | 18.5 | 0.250 | 0.898 | 0.256 | 0.895 | 0.818 |

*PPV* positive predictive value, *NPV* negative predictive value

**Table S5** AUCs for predicting in-hospital mortality among the five scores in patients with MODS and without MODS

|  | **MODS n=150** | | | **Non-MODS n=270** | | |
| --- | --- | --- | --- | --- | --- | --- |
| **Variables** | **AUC** | **95%CI** | ***p* value** | **AUC** | **95%CI** | ***p* value** |
| CMPMIT-ICD10 | 0.8706 | 0.7953-0.9459 | 0.038 | 0.8708 | 0.7780-0.9637 | 0.047 |
| APACHEⅡ | 0.8789 | 0.8069-0.9509 | 0.037 | 0.7936 | 0.6851-0.9021 | 0.055 |
| SOFA | 0.7489 | 0.6427-0.8551 | 0.054 | 0.5646 | 0.3800-0.7492 | 0.094 |
| ISS | 0.6500 | 0.5317-0.7683 | 0.060 | 0.5332 | 0.3540-0.7123 | 0.091 |
| AIS | 0.5689 | 0.4494-0.6883 | 0.061 | 0.4907 | 0.2820-0.6994 | 0.106 |

**Table S6** Best cutoff values, sensitivity, specificity, PPV, NPV, and accuracy of the five scoresin patients with MODS and without MODS

|  | **MODS n=150** | | | | | | **Non-MODS n=270** | | | | | |
| --- | --- | --- | --- | --- | --- | --- | --- | --- | --- | --- | --- | --- |
| **Variables** | **Cutoff value** | **Sensitivity** | **Specificity** | **PPV** | **NPV** | **Accuracy** | **Cutoff value** | **Sensitivity** | **Specificity** | **PPV** | **NPV** | **Accuracy** |
| CMPMIT-ICD10 | 64.5 | 0.867 | 0.783 | 0.500 | 0.959 | 0.800 | 59.0 | 0.818 | 0.842 | 0.180 | 0.991 | 0.841 |
| APACHEⅡ | 24.5 | 0.800 | 0.842 | 0.558 | 0.944 | 0.833 | 15.5 | 1.000 | 0.490 | 0.077 | 1.000 | 0.511 |
| SOFA | 8.5 | 0.667 | 0.717 | 0.370 | 0.896 | 0.707 | 8.5 | 0.182 | 0.942 | 0.118 | 0.964 | 0.911 |
| ISS | 34.5 | 0.467 | 0.875 | 0.483 | 0.868 | 0.793 | 21.5 | 0.455 | 0.668 | 0.055 | 0.966 | 0.659 |
| AIS | 18.5 | 0.300 | 0.883 | 0.391 | 0.835 | 0.767 | 13.5 | 0.364 | 0.780 | 0.066 | 0.967 | 0.763 |

*MODS* Multiple Organ Dysfunction Syndrome,*PPV*positive predictive value, *NPV*negative predictive value

**Table S7**  AUCs for predicting in-hospital mortality among the five scores in patients with ICU LOS≥7d and ICU LOS<7d

|  | **ICU LOS≥7d n=224** | | | **ICU LOS<7d n=196** | | |
| --- | --- | --- | --- | --- | --- | --- |
| **Variables** | **AUC** | **95%CI** | ***p* value** | **AUC** | **95%CI** | ***p* value** |
| CMPMIT-ICD10 | 0.8305 | 0.7544-0.9066 | 0.039 | 0.9195 | 0.8327-1.000 | 0.041 |
| APACHEⅡ | 0.8148 | 0.7242-0.9054 | 0.046 | 0.9216 | 0.855-0.9882 | 0.034 |
| SOFA | 0.5752 | 0.4316-0.7188 | 0.073 | 0.9335 | 0.8570-1.000 | 0.034 |
| ISS | 0.5642 | 0.4134-0.7149 | 0.077 | 0.8822 | 0.8006-0.9639 | 0.042 |
| AIS | 0.6307 | 0.4933-0.7680 | 0.070 | 0.8726 | 0.7765-0.9687 | 0.049 |

*ICU*  Intensive care unit, *LOS* Length of stay

**Table S8** Best cutoff values, sensitivity, specificity, PPV, NPV, and accuracy of five scoresin patients with ICU LOS≥7d and ICU LOS<7d

|  | **ICU LOS≥7d n=224** | | | | | | **ICU LOS<7d n=196** | | | | | |
| --- | --- | --- | --- | --- | --- | --- | --- | --- | --- | --- | --- | --- |
|  | **Cutoff value** | **Sensitivity** | **Specificity** | **PPV** | **NPV** | **Accuracy** | **Cutoff value** | **Sensitivity** | **Specificity** | **PPV** | **NPV** | **Accuracy** |
| CMPMIT-ICD10 | 67.0 | 0.762 | 0.783 | 0.267 | 0.970 | 0.781 | 59.5 | 0.900 | 0.875 | 0.450 | 0.987 | 0.878 |
| APACHEⅡ | 21.5 | 0.810 | 0.729 | 0.236 | 0.974 | 0.737 | 23.5 | 0.800 | 0.926 | 0.552 | 0.976 | 0.913 |
| SOFA | 7.5 | 0.524 | 0.675 | 0.143 | 0.932 | 0.661 | 4.5 | 0.950 | 0.807 | 0.358 | 0.993 | 0.821 |
| ISS | 34.6 | 0.238 | 0.872 | 0.161 | 0.917 | 0.812 | 21.5 | 0.850 | 0.858 | 0.405 | 0.981 | 0.857 |
| AIS | 4.5 | 1.000 | 0.010 | 0.095 | 1.000 | 0.103 | 10.5 | 0.850 | 0.807 | 0.333 | 0.979 | 0.811 |

A Mechanical ventilation


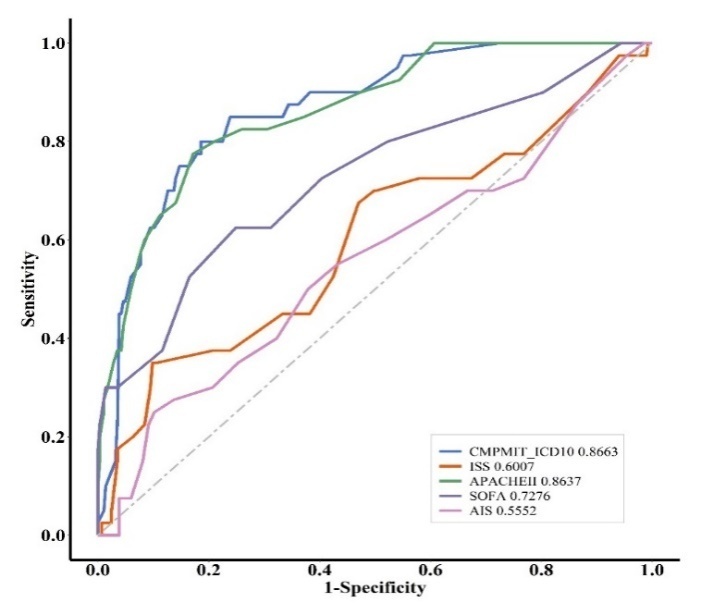


B Comparisons of AUCs


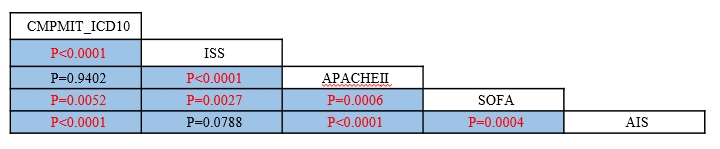


**Fig.S1**Comparisons of areas under the receiver operating characteristic curves.**A**ROC curves for predicting in-hospital mortality in patients with mechanical ventilation. **B**Pair-wise comparisons of AUCs among the five scoresin patients with mechanical ventilation.


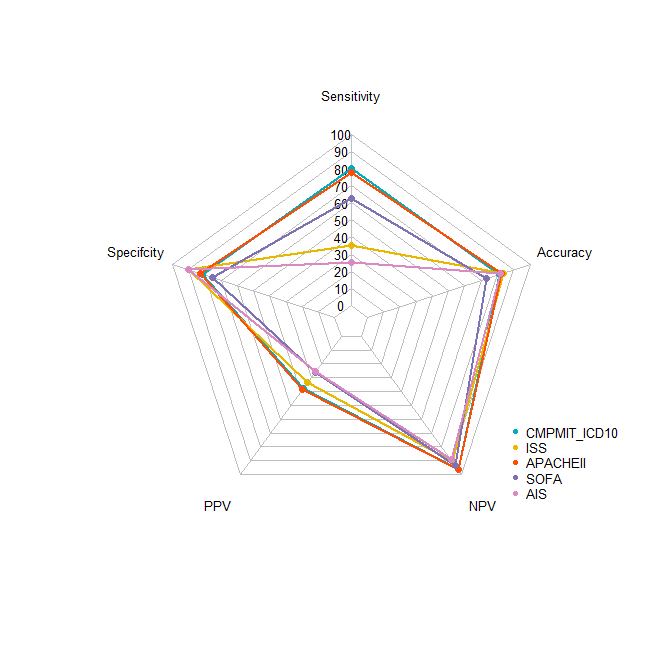


**Fig. S2** Sensitivity, specificity, PPV, NPV, and accuracy for in-hospital mortality of the fivescores in patients with mechanical ventilation.

B non-MODS

A MODS


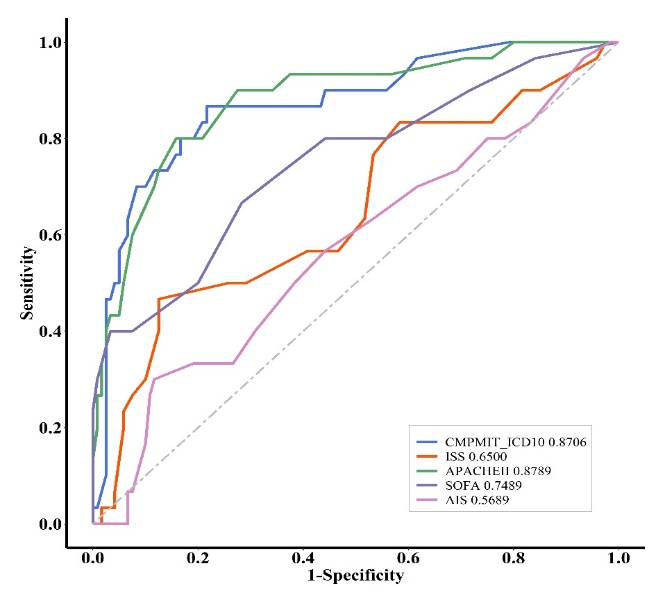

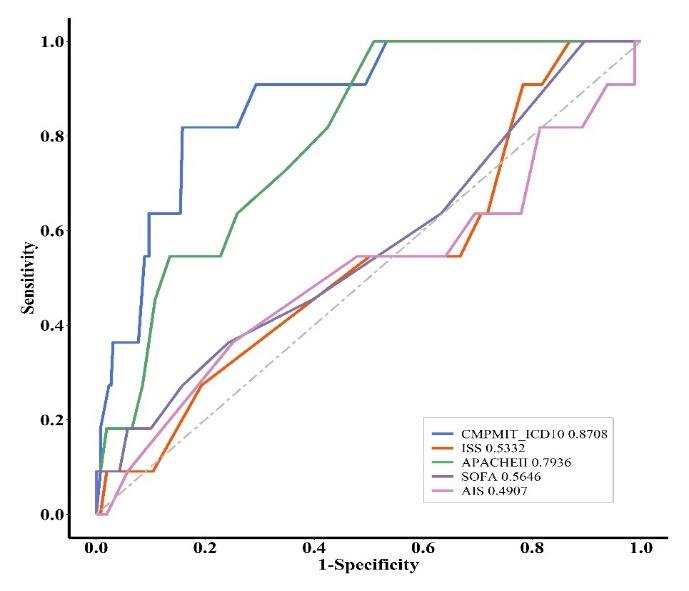


C Comparisons of AUCs


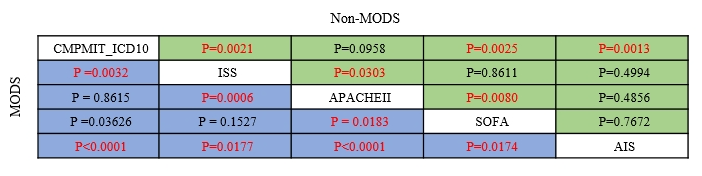


**Fig.S3**Comparisons of areas under the receiver operating characteristic curves.**A**ROC curves for predicting in-hospital mortalityinpatientswithmultiple organ dysfunction syndrome (MODS). **B** ROC curves for predicting in-hospital mortalityinpatientswithoutMODS. **C**Pair-wise comparisons of AUCs for predicting in-hospital mortalityamong the five scores in patients with MODS and without MODS.

B non-MODS

A MODS


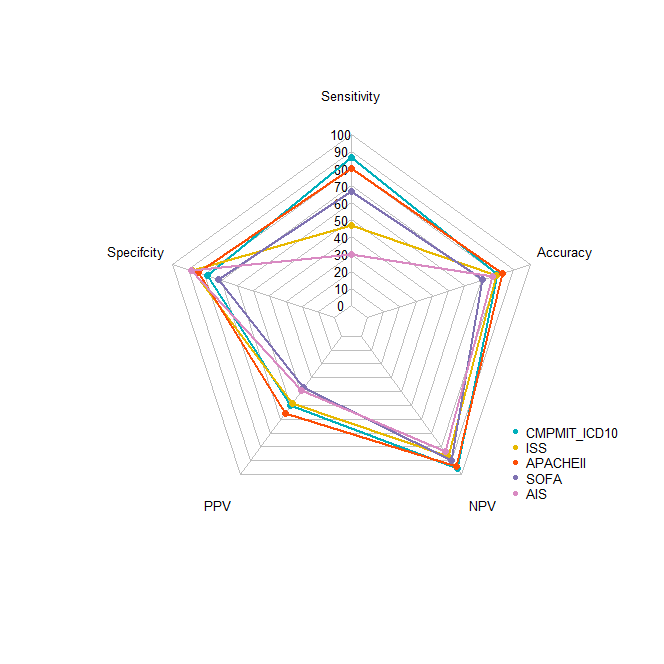

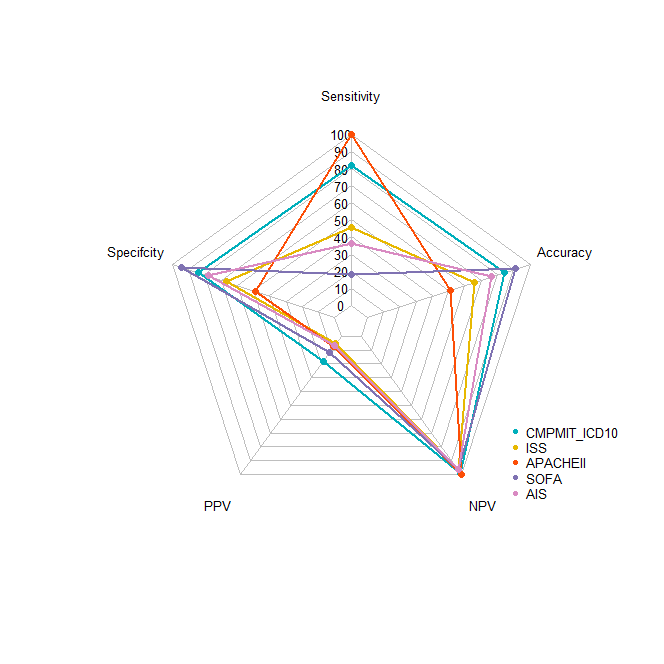


**Fig. S4** Sensitivity, specificity, PPV, NPV, and accuracy for in-hospital mortality of the fivescores in patients with MODS(**A**) and without MODS(**B**)

AICU LOS≥7d

B ICU LOS<7d


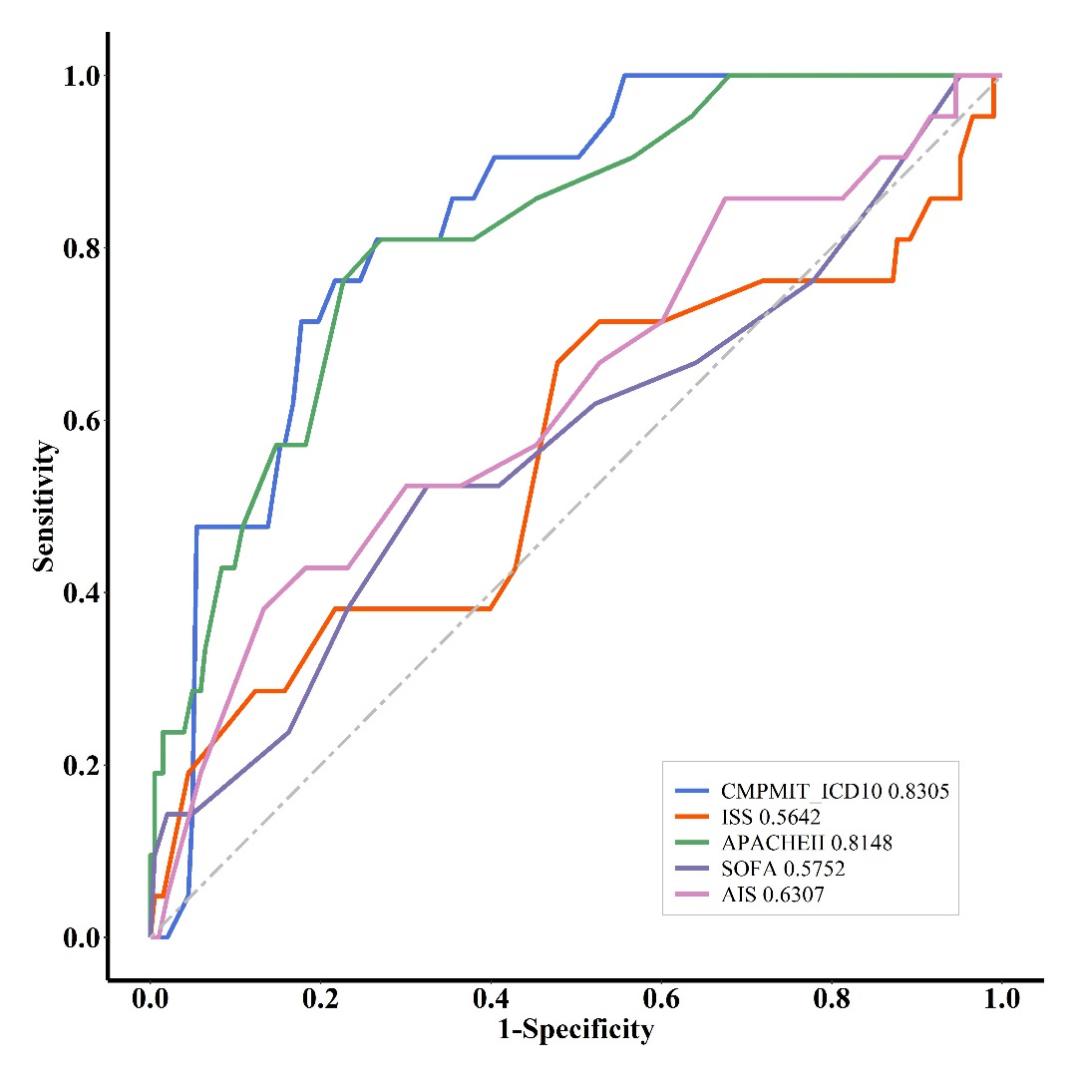

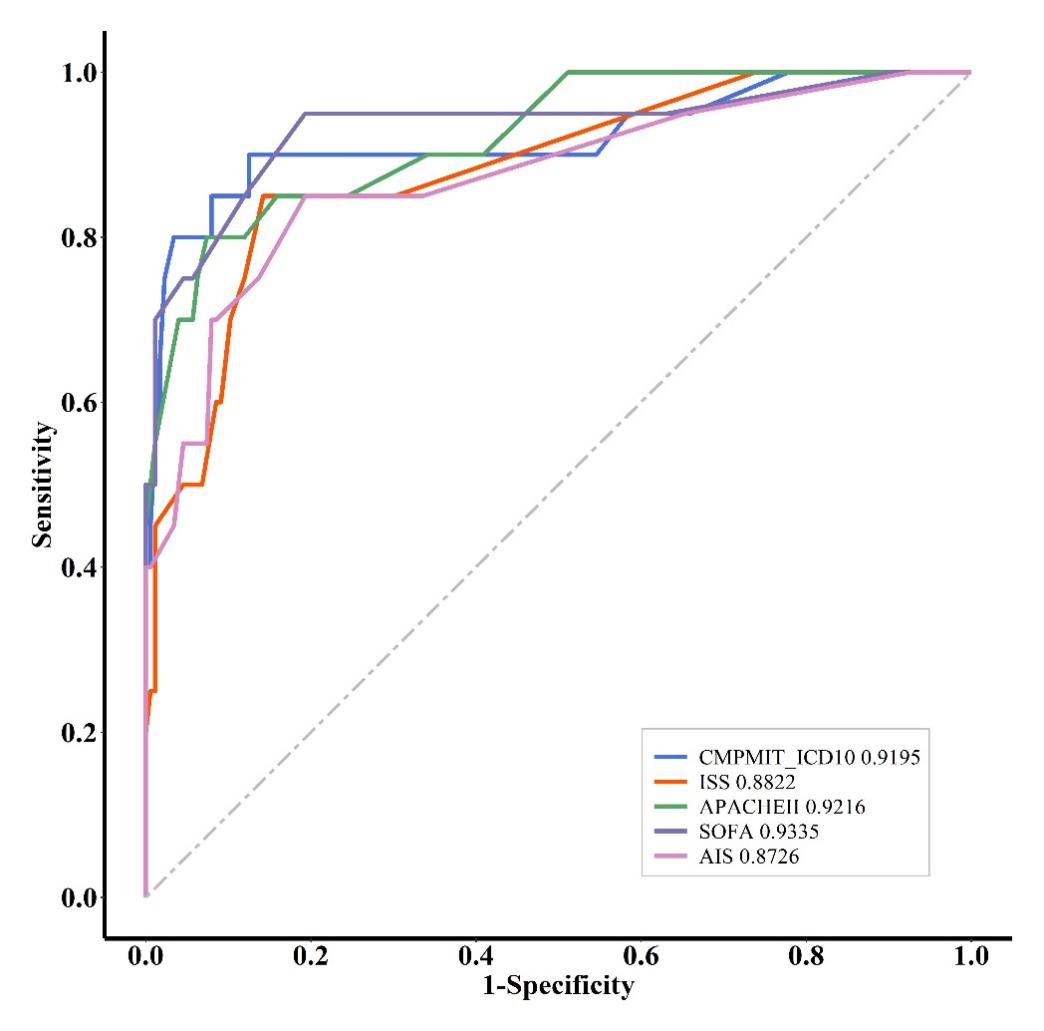


C Comparisons of AUCs


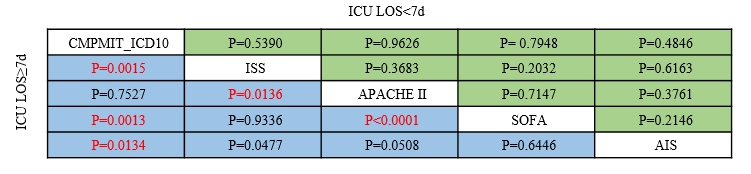


**Fig.S5**Comparisons of areas under the receiver operating characteristic curves. **A**ROC curves for predicting in-hospital mortalityinpatientswithICU length of stay (LOS)≥7d. **B**ROC curves for predicting in-hospital mortalityinpatients with ICU LOS<7d. **C**Pair-wise comparisons of AUCs for predicting in-hospital mortality among the five scores in patients with ICU LOS≥7d and ICU LOS<7d

AICU LOS≥7d

B ICU LOS<7d


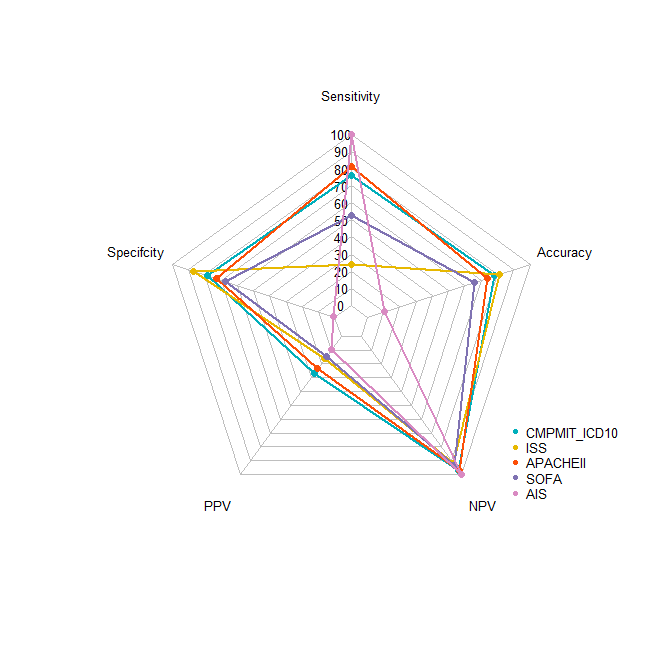

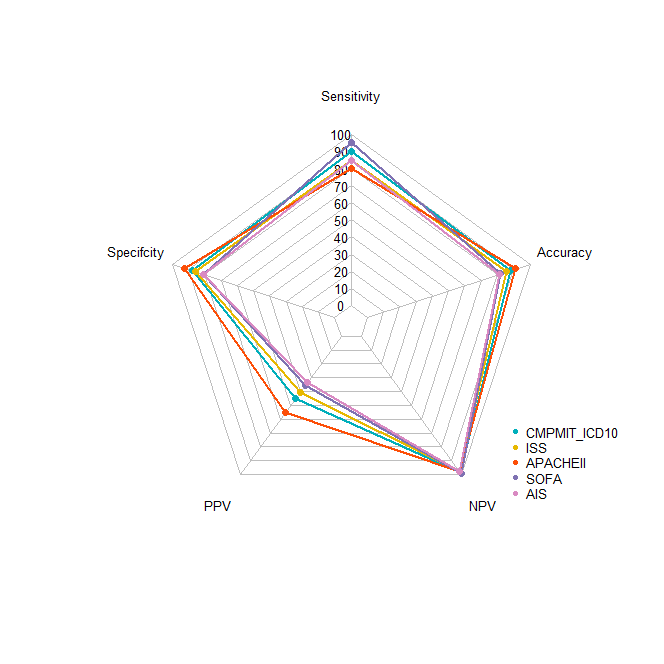


**Fig. S6** Sensitivity, specificity, PPV, NPV, and accuracy for in-hospital mortality of the fivescores in patients with ICU LOS≥7d(**A**) and ICU LOS<7d(**B**)
