# Supplementary material for: Prognostic accuracy of the CMPMIT-ICD-10, APACHE Ⅱ, SOFA, ISS, and AIS for in-hospital death among patients with traumatic hemorrhagic shock
Source: PLoS One. 2026 Feb 5;21(2):e0340159. doi: 10.1371/journal.pone.0340159 (PMC12875474; doi:10.1371/journal.pone.0340159)
Supplement: Attachment — Submitted filename: Review v 1.1.docx [file pone.0340159.s002.docx]

Thank you for inviting me to review the article entitled ‘Prognostic Accuracy of the CMPMIT-ICD10, APACHE-II, SOFA, ISS, and AIS Scoring Scale for In-Hospital Death Among Patients with Traumatic Haemorrhagic Shock**’.**

Serious injuries are the leading cause of death and disability worldwide. Unintentional injuries are ranked as the sixth leading cause of death and the fifth leading cause of moderate to severe disability. The objective of the study was to compare the prognostic performance of the CMPMIT-ICD10 with that of the Acute Physiology and Chronic Health Evaluation II, Sequential Organ Failure Assessment, Injury Severity Score, and Abbreviated Injury Scale for in-hospital mortality in patients with traumatic haemorrhagic shock(THS).

Title:

- The title of the paper reflect the scope of the research.

Abstract:

- The abstract clearly describes the work.

Introduction:

- This section provides a good introduction to the reader's topic.

Methods:

- In the methods section, it is worth adding a description of the elements/parameters of each scale and the range of possible scores on these scales. This can even take the form of a table. This will make it easier for the reader to understand the results of the work.

Results:

- This section is well described.

Discussion:

- Dividing this into subsections, where the results are discussed according to individual scales, would help the reader to understand the topic.

References:

- The references are correctly selected.

I congratulate the authors of the study. The article is very well prepared and clinically useful.
